# The effect of total hysterectomy on sexual function and depression

**DOI:** 10.12669/pjms.313.7368

**Published:** 2015

**Authors:** Sonay Baltaci Goktas, Ismet Gun, Tulin Yildiz, Mehmet Nafi Sakar, Sabiha Caglayan

**Affiliations:** 1 Sonay Baltaci Goktas, Maltepe University, School of Nursing, Surgical Nursing, Istanbul, Turkey; 2 Ismet Gun, Gulhane Military Medical Academy, Haydarpasa Training Hospital, Istanbul, Turkey; 3 Tulin Yildiz, Namik Kemal University, School of Health, Surgical Nursing, Tekirdag, Turkey; 4 Mehmet Nafi Sakar, Suleymaniye Training and Research Hospital, Istanbul, Turkey; 5 Sabiha Caglayan, Medipol University Hospital, Istanbul, Turkey

**Keywords:** Depression, Female, Hysterectomy, Sexual dysfunction, Sexuality

## Abstract

**Background & Objectives:**

To investigate whether the operations of Type 1 hysterectomy and bilateral salpingo-oophorectomy performed for benign reasons have any effect on sexual life and levels of depression.

**Method:**

This is a multi-center, comparative, prospective study. Healthy, sexual active patients aged between 40 and 60 were included into the study. Data was collected with the technique of face-to-face meeting held three months before and after the operation by using the demographic data form developed by the researchers i.e. the Female Sexual Function Index (FSFI) and the Beck Depression Scale (BDS).

**Results:**

In the post-operative third month, there was an improvement in dysuria in terms of symptomatology (34% and 17%, P<0.001), while in FSFI (41.47±25.46 to 34.20±26.67, P<0.001) and BDS (12.87±11.19 to 14.27±10.95, P=0.015) there was a deterioration. For FSFI, 50-60 age range, extended family structure; and for BDS, educational status, not working and extended family structure were statistically important confounding factors for increased risk in the post-operative period.

**Conclusion:**

While hysterectomy and bilateral salpingo-oophorectomy performed for benign reasons brought about short-term improvement in urinary problems after the operation for sexually active and healthy women, they resulted in sexual dysfunction and increase in depression. The age, educational status, working condition and family structure is also important.

## INTRODUCTION

The majority of hysterectomies are performed on benign reasons in order to increase quality of life; nevertheless, it can bring about some post-operative long-term problems such as sexual dysfunction, depression and especially, urinary incontinence.^1^,^2^

During the operation of hysterectomy, particularly in the course of the ablation of cervix, the bilateral inferior hypogastric plexus, which enables the sympathetic and parasympathetic innervations of the sub pelvic region, can sustain injury.^3^ In addition, depending on the lack of uterus among women after hysterectomy and the termination of the capacity of reproduction, the anxiety for no longer having any sex increases the risk of depression, having an impact on the thoughts, social life and partnering communication of women focusing too much on reproduction.^4^ Male partners can also have sexual anxieties after such an operation. In some studies it has been reported that alleviation of sexual problems and anxieties of partners undergoing hysterectomy has a positive effect quality of life of the patients.^5^,^6^ In some studies, it has been pointed out that such operations do not have any effect on the sexual functions of women^7^, while some claim positive effects^8^,^9^ and some others have reported negative effects.^10^,^11^ As a consequence, the majority of contemporary studies are retrospective and the short and long-term effects of hysterectomy on sexual function and depression are still not exactly known.^12^

The aim of this research was to determine, by using the Female Sexual Function Index (FSFI) and the Beck Depression Scale (BDS), whether the operation of Total Abdominal Hysterectomy and Bilateral Salpingo-oophorectomy (TAH+BSO) performed on benign reasons among sexually healthy and active women aged between 40-60 has any effect on sexual life and levels of depression in the post-operative short period.

## METHODS

This study was planned as a prospective and comparative, multi-center one. The patients included into the study were those sexually active and healthy patients, aged between 40-60, who underwent treatment between May 2013 and December 2013 in the Istanbul Gulhane Military Medical Academy, Haydarpasa Training Hospital, Obstetrics and Gynaecology Service, Suleymaniye Training and Research Hospital and Namik Kemal University Medical Faculty Hospital, These were patients with no diagnosis of malignancy, planning to have an operation of Type 1 hysterectomy and bilateral salpingo-oophorectomy and subsequently having this kind of operation. Our study was approved by the Ethics Committee of the Non-Invasive Clinical Trials of GATA Haydarpaşa Training Hospital. All patients were informed about the research; discussions were held about the issues they worry about the operation and the post-operative period. On benign reasons, all patients underwent the operation of Type 1 total abdominal hysterectomy and bilateral salpingo-oophorectomy. In the post-operative six. week, all patients were routinely called in for control, given a briefing on sexual issues, and were encouraged. Either in the pre-operative or post-operative briefing period, the sexual partner was also informed and his anxieties were thus dispelled. The patients who had been under severe depression before the operation or had been using anti-depressant medications or had sexual dysfunction, and those patients who had developed complications during the operation or in the post-operative period, whose partner had a severe illness or had died in the meantime, did not want to continue were excluded.

Demographic features of the women participating in the research were determined through the “Patient Diagnosis Form” prepared by the researchers. Additionally, for the determination of the depression level of women, BDS and for the evaluation of their sexual functions, FSFI scales were used through face-to-face meeting held three months before and after the operation. FSFI were grouped as the following: 0-15 (severe), 16-25 (moderate), 26-35 (mild) and 36 and more (normal); and the Beck Depression Scale was grouped as the following: 0-10 (non), 11-17 (mild), 18-23 (moderate), 24 and more (severe). FSFI was also divided into sub-groups under the headings of satisfaction, lubrication, orgasm, arousal, sexual desire and pain.

### Statistical analysis

Data was evaluated by using SPSS for Windows 15.0 software (Statistical Package for the Social Sciences - SPSS Inc., Chicago, Illinois, USA). Descriptive statistical mean values were presented in terms of standard deviation, frequency and percentage. For statistical analytical categorical changes, the chi-square test was used; for continuous data, the student-t test was used; and for the comparison of dependent qualitative date, the McNemar test was used. Multivariable logistic regression was performed to assess the independence of the associations by adjusting for potential confounding factors. For the purpose of assessing the sexual function of women and determining their level of depression, the categories of age (40-45, 46-50 and 51-60), educational status (primary school or its absence, middle school, high school, associate degree or undergraduate), employment status, family type (nuclear family and extended family) and smoking habits were all used as a potential confounding factor for multivariable logistic regression models. For each potential confounder, we calculated adjusted odds ratios (ORs) and 95% CIs. Results were evaluated in a 95% confidence interval, p<0.05 significance level and p<0.01 P<0.001 advanced significance level. For all comparisons, nominal dual p value was accepted.

## RESULTS

One hundred fifty patients were included into the study. 89 of these patients (59.4%) were in the pre-menopausal period and 61 of them (40.6%) were in the postmenopausal period. The average age of the patients was 46.94±3.86 years; the average marriage duration was 22.40±6.94 years; and average BMI was 26.87±4.14 kg/m^2^. Table-I shows the general demographic features of the patients.

**Table-I:** Demographic features of the patients.

Characters		Frequency	Percentage (%)
Age	40-45age range	65	43.3
	46-50age range	58	38.7
	51-60age range	27	18.0
Educational Status	Literate	12	8.0
	Primary school	44	29.3
	Middle school	28	18.7
	High school	36	24.0
	Associate degree	10	6.7
	Undergraduate	20	13.3
Number of Children	0	8	5.3
	1	23	15.3
	2	64	42.7
	3	39	26.0
	4 and more	16	10.7
Family Type	Nuclear family	107	71.3
	Extended family	43	28.7
Employment Status	Working	79	52.7
	Not Working	71	47.3
Personal House Ownership	Yes	129	86.0
	No	21	14.0
Cigarette smoking	Yes	61	40.7
	No	89	59.3
Alcohol Use	Yes	14	9.3
	No	136	90.7
Sports	Yes	32	21.3
	No	118	78.7

Data are presented as frequency and percentage.

In terms of the symptomatology in the pre-operative and post-operative periods, Table-II shows that there is a statistically significant decrease in urinary problems in the post-operative period (34% and17%, p<0.001, respectively). As for a comparison in terms of FSFI, one can see that there is a statistical difference between the total (41.47±25.46 to 34.20±26.67, p<0.001, respectively) and pre-menopausal sub-groups (42.11±23.83 to 32.52±26.29, p<0.001, respectively) and this difference also continues between sub-groups. And in the menopausal group, there was a difference only in the sub-group of sexual desire (4.3±1.95 to 3.61±1.64, p=0.002, respectively). However, when female sexual functions are grouped as normal and abnormal, there is a difference only in pre-menopausal patients (p=0.031). Table-II indicates that in terms of BDS there is a similar statistical difference between the total (12.87±11.19 to 14.27±10.95, P=0.015, respectively) and pre-menopausal sub-group (P=0.028), but t there was no difference in the menopausal group.

**Table-II:** Symptomatology and female sexual function index of the patients in the preoperative and postoperative periods.

Characters	Preoperative Period	Postoperative Period	P
*Symptoms, n(%)*			
Hot flush and perspiration	75(50)	63(42)	0.203^c^
Sleep problems	57(38)	57(38)	1.00^c^
Urination problems	51(34)	25(17)	<0,001^c^
Muscle and joint disorders	54(36)	50(34)	0,716^c^
FSFI, mean±SD	41.47±25.46	34.20±26.66	<0.001^a^
Satisfaction	7.35±5.07	5.95±5.24	<0.001^a^
Lubrication	8.87±6.11	7.21±6.32	<0.001^a^
Orgasm	6.79±4.70	5.49±4.75	<0.001^a^
Arousal	7.46±4.69	5.93±5.04	<0.001^a^
Sexual desire	4.22±1.82	3.59±1.70	<0.001^a^
Pain	6.77±5.22	6.03±5.49	0.017^a^
Premenopause FSFI, mean±SD	42.11±23.83	32.52±26.30	<0.001^a^
Satisfaction	7.61±4.78	5.89±5.20	<0.001^a^
Lubrication	9.10±5.97	6.81±6.34	<0.001^a^
Orgasm	7.04±4.44	5.18±4.58	<0.001^a^
Arousal	7.45±4.46	5.57±5.14	<0.001^a^
Sexual desire	4.17±1.74	3.57±1.75	<0.001^a^
Pain	6.74±5.01	5.49±5.15	<0.001^a^
Menopause FSFI, mean±SD	40.52±27.85	36.66±27.23	0.19^a^
Satisfaction	6.97±5.48	6.05±5.36	0.082^a^
Lubrication	8.54±6.34	7.80±6.30	0.258^a^
Orgasm	6.43±5.07	5.95±4.00	0.367^a^
Arousal	7.48±5.04	6.44±4.88	0.084^a^
Sexual desire	4.30±1.95	3.61±1.64	0.002^a^
Pain	6.82±5.55	6.80±5.92	0.976^a^
*FSFI, n(%)*			
Normal	100(66.7)	83(55.3)	0.001^b^
Mild	10(6.7)	5(3.3)	
Moderate	2(1.3)	6 (4.0)	
Severe	38(25.3)	56 (37.3)	
*Premenopause FSFI, n(%)*			<0.001^b^
Normal	62(69.7)	47(52.8)	
Mild	8(9.0)	3(3.4)	
Moderate	1(1.1)	5(5.6)	
Severe	18(20.2)	34(38.2)	
*Menopause FSFI, n(%)*			0.564^b^
Normal	38(62.3)	36(59)	
Mild	2(3.3)	2(3.3)	
Moderate	1(1.6)	1(1.6)	
Severe	20(32.8)	22(36.1)	
FSFI abnormal, n(%)	50(33.3)	67(44.7)	0.058^c^
Premenopause	27(30.3)	42(47.2)	0.031^c^
FSFI abnormal, n(%)			
Menopause	23(37.7)	25(41)	0.853^c^
FSFI abnormal, n(%)			

Data are presented as mean±SD and number (percent). FSFI: Female Sexual Function Index;

a Student t test;

b McNemar test;

c chi-square test.

**Table-III:** Depression among the patients in the preoperative and postoperative periods.

Characters	Preoperative Period	Postoperative Period	P
BDS, mean±SD	12.87±11.19	14.27±10.95	0.015^a^
*BDS, n(%)*			
No Depression	77(51.3)	74(49.3)	0.016^b^
Mild	19(12.7)	19(12.7)	
Moderate	20(13.3)	20(13.3)	
Severe	34(22.7)	37(24.7)	
*Premenopause BDS, n(%)*			
No Depression	40(44.9)	41(46.1)	0.028^b^
Mild	14(15.7)	10(11.2)	
Moderate	11(12.4)	10(11.2)	
Severe	24(27)	28(31.5)	
*Menopause BDS, n(%)*			
No Depression	37(60.7)	33(54.1)	0.197^b^
Mild	5(8.2)	9(14.8)	
Moderate	9(14.8)	10(16.4)	
Severe	10(16.4)	9(14.8)	
Depression, n(%)	73(48.7)	76(50.7)	0.817^c^
Premenopausal depression, n (%)	49(55.1)	48(53.9)	0.880^c^
Menopausal depression, n(%)	24(39.3)	28(45.9)	0.583^c^

Data are presented as mean ± SD and number (percent). BDS, Beck Depression Scale;

a Student t test;

b McNemar test;

c chi-square test.

Comparing the existence or absence of depression in the pre-operative and post-operative periods, no difference was observed in any category. Adjusted ORs and 95% CIs of each potential confounder were calculated for the Female Sexual Function Index (FSFI) and the Beck Depression Scale (BDS). When the adjusted odds ration (OR) of the potential confounding factors affecting the existence of female sexual dysfunction and depression, it was educational status at the undergraduate level that was observed to have less frequent in the pre-operative period, [OR:7.32 (95% CI: 1.09-49.10), p=0.040] and in the case of depression it was the 51-60 age range that was observed to have less frequent. In the case of female sexual dysfunction in the post-operative period, extended family type [OR:5.69 (95% CI: 2.26-14.36), p<0.0001] and the 51-60 age range [OR:3.24 (95% CI: 1.05-10.03), p=0.041] were observed more often; and in the case of depression, education status at the levels of primary school [OR:11.40 (95% CI: 1.18-110.01), p=0.035], associate degree [OR:22.18 (95% CI: 1.28-383.52), P=0.033] and undergraduate degree [OR:13.56 (95% CI: 1.03-178.35), P=0.047] were observed less frequent; and working women [OR:4.48 (95% CI: 1.67-12.05), P=0.003] and extended family type [OR:2.57 (95% CI: 1.06-6.23), P=0.036]. Table-IV shows the adjusted ORs for potential confounding factors that were statistically significant for an increased risk of FSFI and BDS.

**Table-IV:** Multivariable logistic regression analysis.

Potential confounding factors		B	p	OR	95% C.I. for OR	
					Lower	Upper
Postoperative FSFI	Smoking	0,27	0,528	1,31	0,57	3,01
	Literate		0,108			
	Primary school	0,64	0,409	1,90	0,41	8,77
	Middle school	1,13	0,210	3,10	0,53	18,10
	High school	1,56	0,084	4,75	0,81	27,73
	Associate degree	1,09	0,307	2,97	0,37	23,88
	Undergraduate	-0,26	0,798	1,30	0,18	9,47
	Working	-0,91	0,066	2,49	0,94	6,60
	Extended family	1,74	<0.001	5,69	2,26	14,36
	40-45_age range		0,025			
	46-50 age range	-0,38	0,373	0,69	0,30	1,58
	51-60 age range	1,18	0,041	3,24	1,05	10,03
	Constant	-1,36	0,167	0,26		
Postoperative BDS	Smoking	-0,02	0,954	1,02	0,44	2,37
	Literate		0,111			
	Primary school	-2,43	0,035	11,40	1,18	110,01
	Middle school	-1,49	0,225	4,42	0,40	48,64
	High school	-2,21	0,068	9,09	0,85	96,92
	Associate degree	-3,10	0,033	22,18	1,28	383,52
	Undergraduate	-2,61	0,047	13,56	1,03	178,35
	Working	1,50	0,003	4,48	1,67	12,05
	Extended family	0,95	0,036	2,57	1,06	6,23
	40-45 age range		0,196			
	46-50 age range	-0,10	0,809	1,11	0,48	2,55
	51-60 age range	-1,10	0,080	3,01	0,88	10,33
	Constant	1,41	0,269	4,12		

ORs, adjusted odds ratios; FSFI, Female Sexual Function Index; BDS, Beck Depression Scale.

## DISCUSSION

Hysterectomy is among the most frequently performed major gynaecological surgical interventions.^1^ Whether with oophorectomy or not, anxieties concerning sexual function in the post-hysterectomy period are questionable and it is emphasized that each partner’s life quality can be affected after such an operation. However, the exact reason underlying the potential sexual dysfunction after hysterectomy could not be explained up until now.^13^,^14^ It is thought that the neural support of the upper vagina is related to orgasm and lubrication and that many nerves in the pelvic region perform their function through a structure known as uterovaginal plexus. However, literature search for the last two decades shows that the ablation or non-ablation of cervix has no effect on sexual function^15^,^16^ and that there is no difference between the techniques of total abdominal hysterectomy, subtotal hysterectomy and vaginal hysterectomy in terms of post-operative sexual activities and sexual problems.^17^ There is also no evidence in relevant literature that supports the possible relation between vaginal length and sexual function.^18^

While many researchers report that there is a measurable advance in the life style and sexual function after simple hysterectomy,^8^,^9^,^19^,^20^ some others point out to negative results.^10^,^11^ In the studies pointing out to positive results, the main reason has been shown as the decrease of dyspareunia^21^, disappearance of pregnancy anxiety, absence of vaginal bleeding and thus the existence of more time for relationship.^7^ And in a few studies, it has been reported, that there is an improvement for the sexual functions of each partner after hysterectomy.^22^ Some small-scale studies have indicated that the sexual well-being after hysterectomy depends upon the relationship between the partners before the operation and physical well-being.^15^ And in a recent review, it has been reported that if hysterectomy is performed under appropriate indications and with an appropriate technique, it would not have any effect upon sexual functions and these claims do not have any scientific premise.^7^ Nonetheless, many of the long-term effects of hysterectomy on sexual function are still unknown.

As for our study, it has been observe that female sexual dysfunction increased after hysterectomy (41.47±25.46 to 34.20±26.67, p<0.001) (Table-II) and that 50-60 age range [OR:3.24 (95% CI: 1.05-10.03), P=0.041]and living within an extended family [OR:5.69 (95% CI: 2.26-14.36), p<0.0001] were the contributing factors (Table-IV). About 59.3% of our patients were in the pre-menopausal group and 40.7% of them were in the menopausal group. In the pre-menopausal group, while there was an increase in FSFI scores not only in the total group (42.11±23.83 to 32.52±26.29, p<0.001) but also in each and every sub-group, in the menopausal group there was a significant difference (in the form of decrease) only in the sub-group of sexual desire (4.3±1.95 to 3.61±1.64, p=0.002) (Table-II). With regard to a comparison in terms of the existence or absence of sexual dysfunction, there was not any difference in the total group and the menopausal group in the post-operative period. However, in the pre-menopausal sub-group, there was an increase in the post-operative period in terms of the existence of sexual dysfunction (30.3% to 47.2%, p=0.031) (Table-II). In fact, these results show not only that sexual life in the early post-hysterectomy period is negatively influenced, but also that this effect is less in menopausal patients. When the pre-operative period and the post-operative period was compared with respect to symptomatology, it was observed that there was a difference only in terms of the urogenital system and unlike what the relevant literature states^2^, it was also observed that there was a statistically significant decrease in urinary problems (34% and 17%, p<0.001, respectively) (Table-II). This can well be the consequence of the disappearance of the urogenital pressure problems brought about by benign conditions and the implementation of prophylactic antibiotic during the operation.

For a woman, hysterectomy not only signals a loss of the capacity of reproduction, but also of sexual function. This is because uterus makes its contraction felt during orgasm.^23^ With the ablation of ovaries, the sudden loss of sex hormones can further increase such anxieties and complaints of depression. The relevant literature points that situations like sexual dysfunctions and decrease in sexual desire after hysterectomy usually leads to a development of depression and that the most common psychiatric problem after hysterectomy is depression.^24^ In Manyonda’s study, related to the issue at hand, it is stated that hysterectomy does not have influence upon psychological well-being, but that a depressive condition in the pre-operative period increases the percentage of depression in the post-operative period.^25^ Table-III shows that depression varies in terms of BDS in the pre-operative and post-operative periods (12.87±11.19 to 14.27±10.95, p=0.015, respectively), but that there is no difference in any category in terms of the comparison between an existence and absence of depression. In addition, while there was a difference in the pre-menopausal group in terms of BDS (P=0.028), there was no such difference in the menopausal group (p=0.197). This can be due to the sudden decrease in sex hormones with the ablation of ovaries.

Our study is not only a prospective study, but also one that includes sexually healthy patients, having no disorder apart from gynaecological pathology, and it consists of isolated cases where patients were excluded in case of any negative situation affecting sexuality in the post-operative short period. One of the major short coming of our study is the possibility of minimal differences originating from the surgical technique, since this study is a multicenter one besides the limited number of cases.

## CONCLUSION

While planning the operation of hysterectomy and bilateral salpingo-oophorectomy on benign reasons in sexually active and healthy women in the pre-menopausal and menopausal groups, potential symptomatology, female sexual dysfunction and depression should be definitely analyzed for their specific factors. While the operation of Type 1 hysterectomy and bilateral salpingo-oophorectomy enables improvement for women in terms of urinary problems in the post-operative short period, it causes sexual dysfunction and depression in women. Before the operation, doctors and nurses must explicitly inform all patients and even their partners about the operation and its potential consequences.
